# BeEAM conditioning regimen is a safe, efficacious and economical alternative to BEAM chemotherapy

**DOI:** 10.1038/s41598-021-93516-x

**Published:** 2021-07-07

**Authors:** Logan Hahn, Hyun Lim, Tanner Dusyk, Waleed Sabry, Mohamed Elemary, Julie Stakiw, Pat Danyluk, Mark Bosch

**Affiliations:** 1 Department of Medicine, University of Saskatchewan, Saskatoon, SK Canada; 2 Department of Community Health and Epidemiology, University of Saskatchewan, Saskatoon, SK Canada; 3 Provincial Hematology and Blood and Marrow Transplant Program, Saskatchewan Cancer Agency, Saskatoon, SK Canada

**Keywords:** Diseases, Medical research, Signs and symptoms, Oncology, Cancer, Stem cells, Adult stem cells, Haematopoietic stem cells, Health care, Health care economics

## Abstract

In many stem cell transplant centres, BCNU, etoposide, cytarabine and melphalan (BEAM) high-dose chemotherapy (HDCT) has been replaced by the more economic and available bendamustine, etoposide, cytarabine, melphalan (BeEAM) regimen. However, there is a paucity of information on the efficacy and safety of BeEAM HDCT. We describe our experience with BeEAM HDCT in terms of safety, efficacy and cost-savings. We compare overall and progression-free survival to a cohort of patients previously transplanted at our institution with the older BEAM regimen. We performed a retrospective chart review of 41 lymphoma patients undergoing BeEAM HDCT at the Royal University Hospital in Saskatoon, Saskatchewan between 2015 and 2019 to elicit regimen safety in the first 100 days post-transplant. Furthermore, we calculated overall and progression-free survival and constructed corresponding Kaplan–Meier curves, comparing the results to a historical cohort of BEAM patients (n = 86). Finally, we conducted an economic analysis using the financials available at our centre’s pharmacy. With regards to BeEAM HDCT, we report a 100-day transplant-related mortality of 2.4%. Additionally, we report acceptable rates of typhlitis (27%), grade III–IV mucositis (4.9%) and grade III–IV nephrotoxicity (2.4%). In terms of overall and progression-free survival, we found no statistical difference between BeEAM and BEAM (*p* = 0.296; 0.762, respectively). Finally, our economic analysis revealed a net savings of $21,200 CAD per transplant when BeEAM is used in replacement of BEAM. The acceptable safety profile of BeEAM and its comparable efficacy to BEAM are encouraging for the perseverance of this cost-effective HDCT regimen.

## Introduction

As a result of the ground-breaking PARMA and CORAL trials, high dose chemotherapy (HDCT) and subsequent autologous stem cell transplantation (ASCT) has become standard of care in the treatment of relapsed and chemotherapy-sensitive Non-Hodgkin’s lymphoma (NHL)^1,2^. Similarly, HDCT and ASCT has proven effective in the treatment of relapsed and resistant Hodgkin’s Lymphoma (HL)^3,4^. Despite the widespread use of HDCT in stem cell transplantation, the various conditioning protocols have seldomly been compared. Notably, there are limited studies comparing HDCT regimens^5^. BEAM (BCNU, etoposide, cytarabine and melphalan) conditioning has been the most widely-used conditioning regimen for the past 30 years^1^. Recently, some centers have discontinued the use of BEAM chemotherapy in favor of BeEAM (bendamustine, etoposide, cytarabine, melphalan) chemotherapy. Additionally, the unavailability of BCNU has prompted a shift to Bendamusine-based regimens^6^. Additionally, there are concerns over BCNU-related pulmonary toxicity^7–11^. Unfortunately, there is a paucity of research comparing BEAM and BeEAM conditioning regimens in terms of safety, efficacy and cost-effectiveness.

Bendamustine was first synthesized in the early 1960s in the former German Democratic Republic^12^. In vitro studies have shown bendamustine to be highly effective in cell lines with dysfunctional apoptotic pathways causing mitotic failure^13^. A phase I–II trial including 77 patients showed bendamustine-containing chemotherapy to be safe and efficacious in the ASCT setting. Importantly, the authors reported a 100-day transplant-related mortality (TRM) of 0%, and 81% of the trial patients achieved complete remission after a median observation of 18 months^12^.

Creatinine elevations after bendamusine-containing HDCT administration has led to concerns over renal toxicity. Several studies have documented nephrotoxicity related to BeEAM chemotherapy, ranging from clinically-insignificant creatinine rises to dialysis necessitation^6,8,14–17^. Interestingly, a study conducted by Noesslinger et al. found that the majority of patients (33/41) experienced a rise in creatinine within a few days of bendamustine administration. Unlike other studies, no therapeutic intervention was required for any of the patients^14^. However, BeEAM-related nephrotoxicity has been demonstrated to have more severe consequences. For instance, a recent study reported that 10% of patients who were administered BeEAM conditioning developed grade III/IV renal toxicity based on Common Terminology Criteria for Adverse Events (CTCAE) v4.0 guidelines, with one of these patients even requiring dialysis^8^.

Cohort studies comparing nephrotoxicity in the conditioning regimens has been similarly inconclusive. A cohort study comparing BEAM and BeEAM safety profiles found a significant difference in nephrotoxicity with 48% of the BeEAM cohort experiencing renal impairment, while this toxicity was only noted in 7% of the BEAM cohort (*p* < 0.001)^17^. A cohort study published in 2018 found the incidence of nephrotoxicity with BeEAM and BEAM to be 12% and 6%, respectively^16^. However, all reported cases of renal impairment constituted grade 1 toxicities. A large cohort study conducted by Frankiewicz et al. found no difference in grade 2–4 nephrotoxicity incidence between BeEAM and BEAM with each group demonstrating only a small risk of renal impairment (1.6% and 0.6%, respectively)^18^.

While there has been concerns over BeEAM-related nephrotoxicity, there is optimism that the regimen has reduced the risk of IPS^8,12^. This represents a significant advantage of the conditioning regimen, as IPS is associated with a higher rate of TRM and shorter progression-free survival (PFS) and overall survival (OS)^9^. HDCT regimens containing BCNU have been shown to cause IPS at incidences of 1–64%, thus presenting a significant drawback of BEAM therapy^19–21^. A hypothesized advantage of BeEAM chemotherapy is a lower risk of IPS. For instance, a recent study conducted by Gilli et al. demonstrated that IPS is a possible, albeit rare, consequence in BeEAM conditioning^8^.

Current studies have produced widely varying results with regards to the safety and toxicological profile of BeEAM HDCT. Clearly, there is a need to evaluate the safety of BeEAM conditioning, with particular attention to nephrotoxicity and IPS. In this retrospective, single-center study we document our experience with BeEAM conditioning in terms of safety, efficacy and economics.

## Materials and methods

### Patients

In this single-center, retrospective chart review, we analyzed consecutive patients with Hodgkin’s lymphoma (n = 7) or Non-Hodgkin’s lymphoma (n = 34) who have been treated with BeEAM HDCT followed by ASCT between 2015 and 2019 at the Royal University Hospital in Saskatoon, Canada. These patients were followed and charts were reviewed through the Provincial Hematology and Blood and Bone Marrow Transplant Program at the SCA. All patients signed consent prior to proceeding with ASCT. Furthermore, the BeEAM cohort was compared to a previously studied cohort of patients undergoing BEAM ASCT (n = 86) at the aforementioned institution for the purpose of survival analysis. BEAM transplants occurred between 2009 and 2016, as the BeEAM protocol became standard of care at our institution in 2015. This study was approved by the local ethics committee and the Saskatchewan Cancer Agency in accordance with the relevant guidelines and regulations. Detailed patient characteristics can be found in Table 1.

**Table 1:** Patient characteristics.

Patients	BeEAM	BEAM	*p* value^a^
	n = 41	n = 86	
Age, mean, SD	55 (12)	51 (13)	0.123
**Age groups (n, %)**			
≤ 60	24 (58.5)	61 (70.9)	0.165
> 60	17 (41.5)	25 (29.1)	
**Sex (n, %)**			
Female	10 (24.4)	36 (41.9)	0.055
Male	31 (75.6)	50 (58.1)	
**Diagnosis (n, %)**			
Hodgkin lymphoma/nodular sclerosis	7 (17.1)	28 (32.6)	0.068
All others	34 (82.9)	58 (67.4)	
**Total deaths (n, %)**			
Yes	8 (19.5)	27 (31.4)	0.161
No	33 (80.5)	59 (68.6)	
**Deaths 3 years after ASCT (n, %)**			
Yes	8 (19.5)	18 (20.9)	0.853
No	33 (80.5)	68 (79.1)	
**Total relapsed patients (n, %)**			
Yes	8 (19.5)	28 (32.6)	0.127
No	33 (80.5)	58 (67.4)	
**Relapsed patients after ASCT (n, %)**			
Yes	8 (19.5)	23 (26.7)	0.375
No	33 (80.5)	63 (73.3)	

^a^Chi-square test: ASCT autologous stem cell transplantation.

### Treatment

BEAM: 300 mg/m^2^ BCNU was given on day − 6. On days − 5 to − 2, 400 mg/m^2^ cytarabine was administered every 12 h as a 30-min infusion in 100 mL 0.9% NaCl. On days − 5 to −  2, 200 mg/m^2^ etoposide was administered once daily as a 120 min infusion in 1000 mL 0.9% NaCl. Melphalan at 140 mg/m^2^ was administered as a single 60 min infusion in 1000-mL 0.9% NaCl. A minimum of 2.0 × 10^6^ CD34 + cells/kg body weight were reinfused on day 0.

BeEAM: Bendamustine at 200 mg/m^2^ was given as a single 1-h infusion in 1000 mL in 0.9% NaCl on days − 7 and  − 6. On days  − 5 to  − 2, 200 mg/m^2^ cytarabine was administered every 12 h as a 30-min infusion in 100 mL 0.9% NaCl. On days − 5 to  − 2, 200 mg/m^2^ etoposide was administered once daily as a 120 min infusion in 1000 mL 0.9% NaCl. Melphalan at 140 mg/m^2^ was administered as a single 60 min infusion in 1000-mL 0.9% NaCl. A minimum of 2.0 × 10^6^ CD34 + cells/kg body weight were reinfused on day 0.

All patients on BEAM and BeEAM received weight-adapted G-CSF (filgrastim 300mcg if ≤ 70 kg or 480mch if ≥ 70 kg) starting at day + 7 until neutrophils exceeded 1.0 g/L. As per institutional protocol, patients received antiviral (oral valacyclovir 500 mg twice daily) and antifungal prophylaxis (oral fluconazole 200 mg once weekly and oral sulfamethoxazole/ trimethoprim 800/160 mg three times per week). Antibiotic prophylaxis was not used. Patients were transfused with platelets when platelet counts dropped below 10 × 10^9^/L. Patients received red cell transfusions at a hemoglobin threshold of 70 g/L. Patients were hospitalized at the initiation of HDCT and remained in-hospital until hematologic and adequate performance status was achieved.

### Measurements and definitions

Nephrotoxicity was defined according to the Common Terminology Criteria for Adverse Events (CTCAE) v5.0 guidelines. The institutional upper limit of normal for creatinine (104 µmol/L) was used to define recovery at discharge. Typhlitis was only reported if it was confirmed via CT assessment. OS was defined as the time from ASCT to death. PFS was defined as time from ASCT to first relapse/progression, death, or last follow-up, whichever occurred first. Neutrophil engraftment was defined as the first day of three consecutive days where the neutrophil count (absolute neutrophil count) was 500 cells/mm^3^ (0.5 × 109/L) or greater. Platelet engraftment was defined as a platelet count of 20,000/mm^3^ (20 × 109/L) unsupported by a platelet transfusion (> 3 days post-transfusion).

### Statistical analysis

Descriptive analysis was performed to summarize the data. Mean and standard deviation estimates of each continuous variable were calculated separately and compared between HDCT regimens using Student’s *t* tests. For categorical variables, proportion estimates were calculated separately and compared between BEAM and BeEAM using a χ^2^ or Fischer’s exact test when appropriate. The primary endpoints analyzed were progression free survival (PFS) and overall survival (OS). We used the Kaplan–Meier method to understand the survival distribution and to produce survival curves for PFS and OS for both HDCT regimens. Then, we compared the survival distributions between the two groups to determine equivalency using log-rank test. Univariable and multivariate Cox regression models were used to analyze each HDCT and multiple risk factors for OS and PFS separately. The results are given with a hazard rate (HR), confidence interval (CI) of 95% and *p* value. Statistical analyses and associated figures were generated with the Statistical Package for the Social Sciences (SPSS) Version 26 (SPSS Inc. Armonk, NY: IBM Corp.). Statistical significance was defined by an alpha level of *p* ≤ 0.05.

### Economical analysis

Our economic analysis was based on comparison of the list pharmaceutical costs. Only the direct cost of chemotherapy agents was analyzed, and ancillary costs were not factored in the analysis.

### Ethics approval

The following study was approved by the local ethics committee at the Saskatchewan Cancer Agency.

### Consent to participate

Data transfer agreements were signed with the Saskatchewan Cancer Agency.

### Consent for publication

Approval for publication was granted by the Saskatchewan Cancer Agency. The results and conclusions presented here represent the work of the authors and no endorsement by the SCA or by any third party is to be inferred.

## Results

### Engraftment, transfusions and hospitalization (BeEAM patients)

The median time to platelet engraftment was 12.6 days (range: 7–19 days), whereas median time for neutrophil engraftment was 11.7 days (9–15 days) for BeEAM HDCT patients. The median number of platelet transfusions was 4.3 units (range: 2–9 units) per admission for BeEAM ASCT. The median number of packed red blood cell (pRBC) transfusion units was 2.3 units (range: 0–5 units). Interestingly, 53.7% of BeEAM patients required less than two pRBC transfusions. The median number of days of admission to hospital for BeEAM transplantation was 24.2 days (range: 18–33 days) (Table 2).

**Table 2:** BeEAM toxicities.

Patients	BeEAM
	n = 41
Death prior to Day 100	2.4% (n = 1)
**Gastrointestinal toxicity**	
Mucositis	88% (n = 36)
Grade I–II	n = 34
Grade III–IV	n = 2
Typhlitis	27% (n = 11)
**Renal toxicity**	
All grades	32% (n = 13)
Grade I–II	n = 12
Grade III–IV	n = 1
**Cardiac toxicity**	
New onset atrial fibrillation	15% (n = 6)
Myocardial infarction	2.4% (n = 1)
**Infectious complications**	
Febrile neutropenia	100% (n = 41)
Septic shock	12.2% (n = 5)
ICU admissions	2.4% (n = 1)
**Bacterial infections**	56% (n = 23)
Bacteria gram + ve (%)	66.7%
Bacteria gram − ve (%)	33.3%
Viral infections	14.6 (n = 6)
Fungal infections	2.4% (n = 1)
No organism identified	29.3% (n = 12)
**Transfusion and engraftment**	
Median days to platelet engraftment	12.6 (7–19)
Median days to neutrophil engraftment	11.7 (9–15)
Median platelet transfusions	4.3 (2–9)
Median red blood cell transfusions	2.3 (0–5)
**Hospitalization**	
Median days in hospital	24.2 (18–33)

### Toxicities (BeEAM patients only)

Thirteen BeEAM patients (32%) experienced nephrotoxicity according to CTCAE v5.0 guidelines. Twelve of these patients experienced grade I–II toxicity, while one patient suffered grade III renal toxicity. Mean baseline creatinine for patients experiencing nephrotoxicity was 87.6 µmol/L (range: 51–128 µmol/L), compared to 76.5 µmol/L (range: 38–157 µmol/L) in patients who did not experience nephrotoxicity. All patients were treated expectantly with no patient requiring dialysis. Interestingly, nine of these thirteen patients had elevated creatinine levels at discharge, indicating some persistence of renal impairment. Thirty-six patients (88%) experienced documented mucositis. Thirty-four of these patients suffered grade I-II mucositis, whereas two patients experienced grade III mucositis. Eleven patients (27%) experienced CT-confirmed typhlitis, and all patients were treated with IV antibiotics and bowel rest. Six patients (15%) experienced new-onset atrial fibrillation during the course of their admission for ASCT (Table 2).

### Infections (BeEAM patients)

All patients undergoing BeEAM ASCT experienced at least one episode of febrile neutropenia. No organism was identified in twelve (29.3%) of these aforementioned patients. 23 patients had a documented bacterial infection. Among documented bacterial infections, 66.7% of isolated species were gram positive and 33.3% were gram negative species. Six patients (14.6%) had documented viral infections. Five of these viral infections were with common respiratory viruses (rhinovirus, coronavirus, parainfluenza) and one patient experienced cystitis related to BK virus reactivation. One patient (2.4%) experienced a fungal infection in the form of aspergillus. Five patients (12.2%) experienced septic shock, with one of these patients being admitted to the ICU and another requiring consultation from ICU. No patients died from infectious complications during ASCT (Table 2).

### Survival

One sudden death occurred in the BeEAM cohort. This death occurred on day + 14 from stem cell infusion and was felt to be due to a cardiac arrest in a patient with known cardiac disease, yielding a TRM of 2.4%. The 3-year OS was 78.1% for BEAM, and 71.0% for BeEAM. The 3-year PFS was 71.3% for BEAM, and 74.1% for BeEAM. Our 3-year calculation of OS and PFS showed no significant differences between the two HDCT regimens (Table 3). Interestingly, multivariate analysis of covariates such as age, sex and diagnosis yielded no significant effect on OS or PS at 3-year follow-up. Multivariate analysis revealed no significant difference in OS or PFS between the two conditioning regimens (*p* = 0.372, 0.801; respectively) (Tables 4, 5).

**Table 3:** Probability of 3-year survival.

	Number of patients	Overall survival (OS)				Progression free survival (PFS)			
		3-Year probability (%)	95% CI		*p* value	3-Year probability (%)	95% CI		*p* value
			Lower	Upper			Lower	Upper	
Whole cohort	127	76	67.8	84.2	–	71	62.2	79.8	–
BEAM	86	78.1	69.08	87.12	0.296	71.3	61.3	81.3	0.762
BeEAM	41	71	52.4	89.6		74.1	58.2	90.0	
**Age**									
≤ 60	85	75.3	65.5	85.1	0.718	68.1	57.1	79.1	0.433
> 60	42	76.6	61.1	92.1		77.3	63.2	91.4	
**Sex**									
Female	46	76.3	63.4	89.2	0.815	68.5	54.2	82.8	0.953
Male	81	75.6	65.0	86.2		72.6	61.6	83.6	
**Dx**									
Hodgkin lymphoma/nodular sclerosis	92	71.9	61.5	82.3	0.267	70.5	59.9	81.1	0.671
All others	35	84.7	72.4	97.0		71.4	55.5	87.3

**Table 4:** Overall survival: multivariate Cox regression model.

Time to death	HR	95% CI		*p* value
		Lower	Upper	
**Regimen**				
BEAM				
BeEAM	1.483	0.625	3.518	0.372
**Age**				
≤ 60				
> 60	0.733	0.33	1.63	0.446
**Sex**				
Female				
Male	0.995	0.494	2.001	0.988
**Dx**				
All others				
Hodgkin lymphoma/nodular sclerosis	0.603	0.262	1.391	0.236

**Table 5:** Progression-free survival: multivariate Cox regression model.

Time to Relapse	HR	95% CI		*p* value
		Lower	Upper	
**Regimen**				
BEAM				
BeEAM	1.114	0.482	2.574	0.801
**Age**				
≤ 60				
> 60	0.677	0.306	1.494	0.334
**Sex**				
Female				
Male	0.942	0.472	1.877	0.864
**Dx**				
All others				
Hodgkin lymphoma/nodular sclerosis	0.756	0.34	1.684	0.494

### Economic analysis

Our economic analysis, which used local pharmacy data, revealed tremendous cost benefits of BeEAM HDCT in comparison to BEAM HDCT. At our center in Saskatchewan, Canada, the cost of a BeEAM ASCT is $12,181 CAD, whereas the cost of a BEAM ASCT is $33,381 CAD. This equates to a net savings of $21,200 CAD per transplant. The introduction of BeEAM at our centre in 2015 has resulted in a total savings of approximately $890,000 CAD from 2015 to 2019.

## Discussion

There is a paucity of research comparing HDCT regimens in the context of stem cell transplantation. The present study depicts our institution’s experience with BeEAM chemotherapy in terms of toxicity, efficacy and cost in reference to the previous BEAM regimen. To our knowledge, it is the first study to explore both the clinical and financial considerations associated with this widespread HDCT transition. This single-centre study suggests several reasons for the perseverance of BeEAM as the conditioning regimen of choice at our institution.

Our study found an incidence of grade I–IV renal toxicity of 32% in the BeEAM HDCT cohort. This is consistent with previous retrospective studies, however the incidence of nephrotoxicity varies widely in the literature from 1.6 to 48%^6,15–18^. The incidence of BeEAM nephrotoxicity reported here is higher than that reported for BEAM in previous cohort studies^16–18^. However, the significance of our findings should be interpreted cautiously as all patients with renal impairment were treated expectantly and renal function did not delay the HDCT/ASCT regimen for any patient followed. Additionally, it has been reported in the literature that renal insufficiency during ASCT does not impact hematopoietic stem cell collection, affect mucositis incidence, delay engraftment or increase transfusion requirements^22,23^. Also, the renal toxicity experienced by ASCT patients may not be fully imputable to bendamustine, as patients undergoing ASCT are frequently exposed to other nephrotoxic agents such as vancomycin. Despite this, precautions such as the provision of adequate hydration and the avoidance of bendamustine dose escalations beyond 200 mg/m^2^/day are generally recommended^14,16^.

The prevalence of oral mucositis amongst BeEAM patients was 88%, which is concordant with other studies^6,8,10^. Interestingly, we report relatively low rates of grade III–IV oral mucositis, with only 2 patients experiencing grade III toxicity, whereas a recent study conducted by Visani et al. reported that approximately 25% of patients receiving BeEAM HDCT experienced grade III–IV mucositis^12^. Another study reported the incidence of grade III–IV oral mucositis to be 42.9% in NHL patients receiving the older BEAM chemotherapy^24^. Thus, these results are suggestive of a favourable toxicological profile with regards to oral mucositis. Interestingly, our institutional mucositis protocol only involves club soda mouthwashes four times a day.

Typhlitis is an important toxicological consideration with associated mortality rates as high as 50% reported in the literature^25^. Eleven patients (27%) developed CT-confirmed typhlitis in the present study. Diagnostic criteria and incidences of typhlitis vary amongst comparable studies with incidences of 17–35% reported in the literature^6,12,17^. Cohort studies comparing the digestive toxicity of BeEAM and BEAM have also garnered mixed results^16–18^. None of the patients who developed typhlitis required surgery. We suspect typhlitis rates are high at our centre as we do not employ prophylactic antibiotics, due to hospital antibiotic stewardship measures.

Additionally, we report 6 cases of new-onset atrial fibrillation within our BeEAM cohort. This finding is congruous with previous studies which report a small to moderate risk of BeEAM-associated cardiotoxicity^6,8,14^. Additionally, one cardiac-related death was reported in the present study. All cases of new-onset atrial fibrillation were managed conventionally with beta-blockers. It is important to note that cardiotoxicity is not exclusive to the BeEAM conditioning regimen. For instance, a large cohort study conducted by Robinson et al. reported 3 cases of fatal cardiac toxicity associated with BEAM chemotherapy^26^.

Within our small BeEAM cohort, 56% of patients experienced a culture-positive bacterial infection. However, this is consistent with previous studies which report BeEAM-associated bacteremia rates ranging from 24 to 64%^8,16,17^. While no patient in our BeEAM cohort died of infectious complications, one patient did require treatment in the ICU. It is important to note that cohort studies comparing the toxicity of BeEAM and BEAM have not yielded significant differences between the conditioning regimens in terms of bacteremia rates^16,17^.

No patients in our BeEAM cohort developed IPS, which is a dreaded transplant complication. This may reflect a potential advantage over BCNU chemotherapies which carry a known risk of IPS^19–21^. However, the research remains uncertain as to the true risk of BCNU with regards to IPS. A recent study, for instance, found that BCNU doses of 300 mg/m^2^ were only associated with a 0.7% incidence of pulmonary toxicity^27^. Thus, the cost utility of mitigating IPS is unknown at this time.

**Figure 1:**
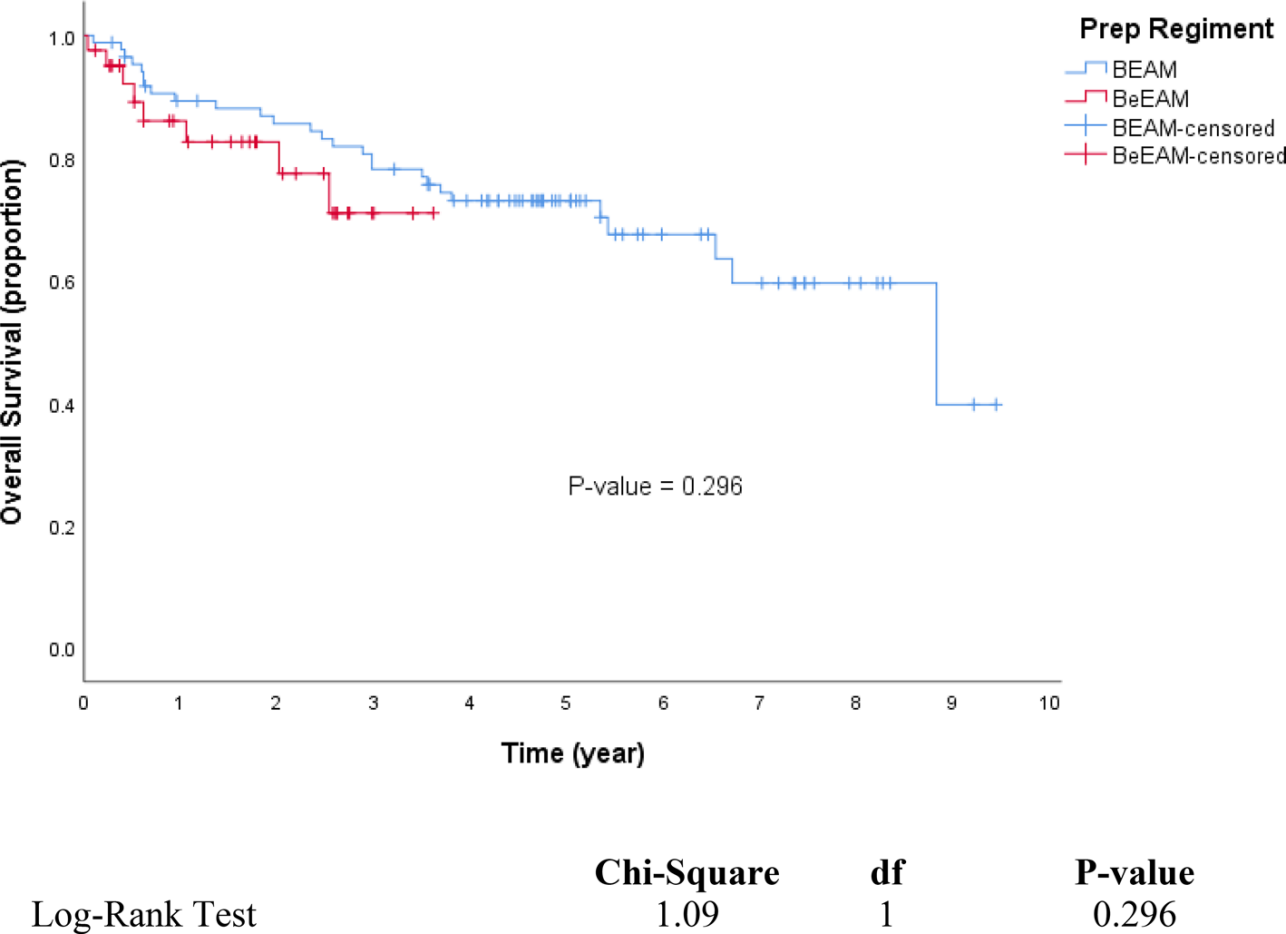
Kaplan–Meier analysis of overall survival. Overall survival (OS) in patients who underwent an ASCT with BEAM or BeEAM. *ASCT* autologous stem cell transplantation, *BEAM* BCNU, etoposide, cytarabine, melphalan, *BeEAM* bendamustine, etoposide, cytarabine, melphalan. *p* values were determined using the log-rank test.

**Figure 2:**
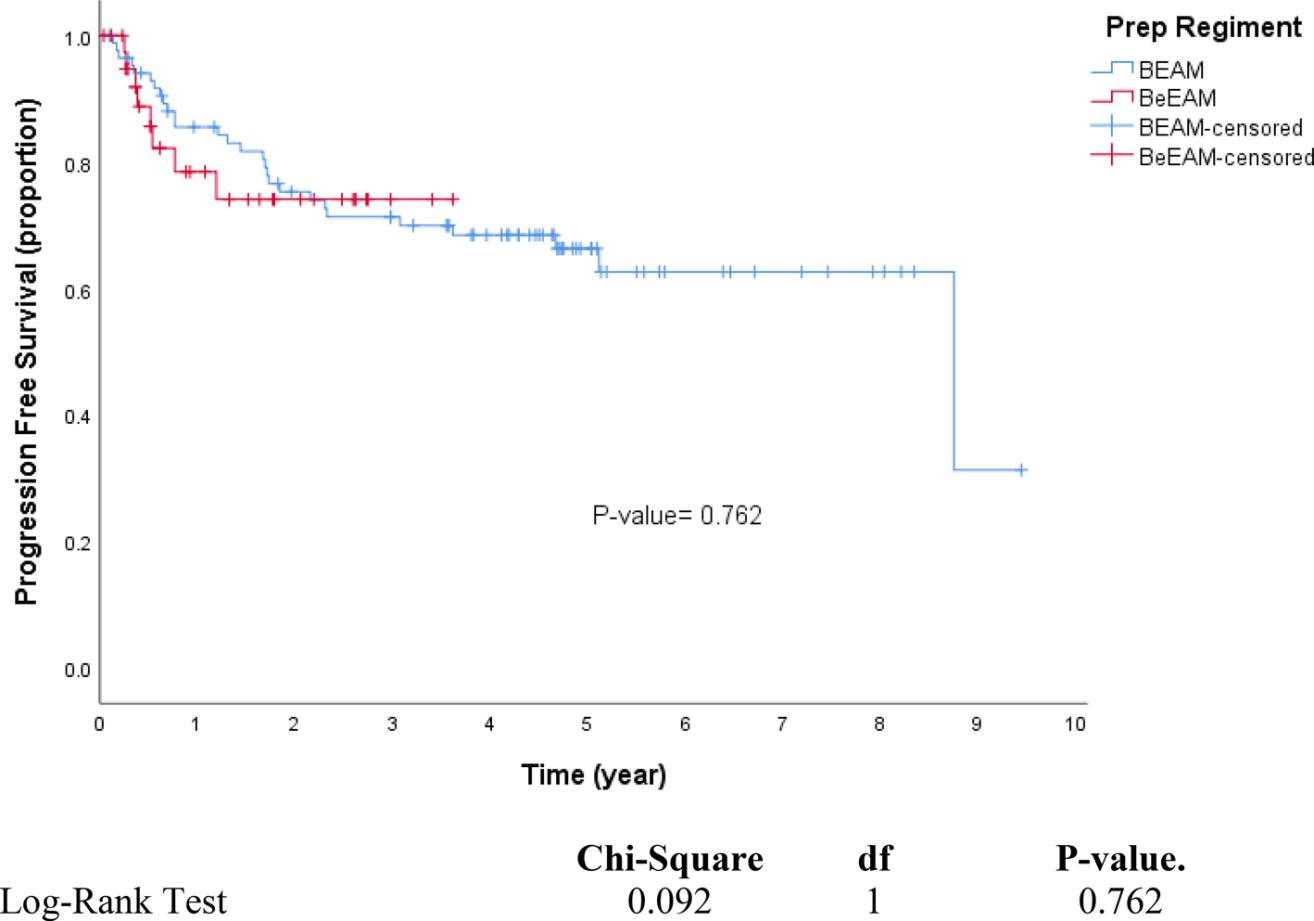
Kaplan–Meier analysis of progression-free survival. Progression-free survival in patients who underwent an ASCT with BEAM or BeEAM. *ASCT* autologous stem cell transplantation, *BEAM* BCNU, etoposide, cytarabine, melphalan, *BeEAM* bendamustine, etoposide, cytarabine, melphalan. *p* values were determined using the log-rank test.

We constructed Kaplan–Meier curves comparing OS and PFS in our BeEAM cohort and a historical BEAM cohort from our centre (Figs. 1, 2). We found no difference in OS and PFS between the conditioning regimens in multivariate analysis (*p* = 0.372, 0.801; respectively). This is consistent with other cohort studies^16–18^. We report a 100-day TRM of 2.4% in our BeEAM cohort, indicating acceptable safety and efficacy of the conditioning regimen. Interestingly, our multivariate analysis did not yield any significant differences with respect to age, sex or diagnosis of the patient undergoing transplantation. Perhaps this is indicative of widespread acceptability of the HDCT.

Unfortunately, the present study is unable to directly compare the toxicological profiles of each HDCT regimen, as we do not presently have access to acute transplant toxicity data for the BEAM cohort. Another disadvantage of the present study is a relatively short duration of follow-up associated with BeEAM HDCT due to its recent enrolment at our centre. Furthermore, our cost analysis was limited to the raw cost of the chemotherapies at our institution. Therefore, ancillary costs related to administration of the HDCTs were not included but not expected to be significantly different between cohorts.

Our data confirms that BeEAM chemotherapy has adequate PFS and OS, an acceptable safety profile and marked cost-savings in comparison to BEAM chemotherapy. Based on this study, BeEAM chemotherapy will remain standard of care at our institution. Our centre’s experience with the BeEAM regimen suggests that the small risk of often clinically-insignificant nephrotoxicity is offset by tremendous cost-effectiveness. However, larger, prospective trials are necessary to fully elucidate the benefits and risks of BeEAM chemotherapy.

## Data Availability

Not applicable.
